# Vascular disease as a cause of death in patients with severe disability due to osteoarthritis and rheumatoid arthritis

**DOI:** 10.1186/s40064-015-1108-2

**Published:** 2015-07-08

**Authors:** Ann Marie Smith, Liz Lingard, Peta Heslop, Janine Gray, David J Walker

**Affiliations:** Rheumatology Department, Room 22, Musculoskeletal Unit, The Newcastle upon Tyne Hospitals NHS Foundation Trust, The Freeman Hospital, Newcastle upon Tyne, NE7 7DN UK; North East Quality Observatory System (NEQOS), 1st Floor, Ridley House, Henry Street, Gosforth, Newcastle upon Tyne, NE3 1DQ UK; Clinical Trials Office, Education Centre, North Tyneside General Hospital, Rake Lane, North Shields, Tyne & Wear, NE29 8NH UK; Clinical Trials Research Unit, Leeds Institute of Clinical Trials Research, University of Leeds, Leeds, LS2 9JT UK

**Keywords:** Rheumatoid arthritis, Osteoarthritis, Knee surgery, Cardio-vascular disease

## Abstract

**Objectives:**

The mechanism of the increased risk of cardiovascular disease in rheumatoid arthritis (RA) remains uncertain. We had the opportunity to compare the causes and ages of death in a population of osteoarthritis (OA) and RA patients who had had similar lower limb disability.

**Methods:**

Death certificates were sought for a population of OA and RA patients who had had knee joint replacements performed by a single orthopaedic surgeon over a 10 year period with a minimum follow up period of 18 years. Primary cause of death was assigned by a blinded clinician and compared between the populations. Competing risk analysis was used to compare RA and OA populations for cardiovascular deaths.

**Results:**

The total population was 607 (294 OA; 313 RA). 85% (249) of the OA and 79% (246) of the RA patients had deceased at the time of study in 2008. 85% of the death certificates were found. The RA patients were operated an average of 7.5 years younger and also died 7.5 years younger. The causes of death were similar in the two populations. The ages at death were consistently and similarly older for the OA group for all causes of death. There was a 9% increased risk of cardiovascular death in the RA group but this was not statistically different from the OA group.

**Conclusions:**

OA and RA patients, controlled for lower limb disability, have similar causes of death including cardiovascular disease. However, the RA patients died significantly younger. Cause of death is likely to be related to things that OA and RA share, such as disability and some treatments e.g. NSAIDs, whereas age at death relates to differences, such as age of onset and inflammation.

## Background

The association of rheumatoid arthritis (RA) with increased vascular disease is well established (Kitas et al. 2001; Sattar et al. 2003). The mechanism of this risk is less certain with studies of traditional factors showing conflicting results (McEntegart et al. 2001; del Rincon et al. 2001; Maradit-Kremers et al. 2005). There is stronger association with higher disease activity and disability, and extra-articular disease (Peltomaa et al. 2002) but these are interrelated. In a recent study from our area, cardiovascular disease was found to be a greater cause of death in longstanding RA patients when compared to their siblings, but not when compared to similarly longstanding osteoarthritis (OA) patients (Kumar et al. 2007). This suggested that the risk factors for vascular disease were shared by OA and RA rather than being familial. We were presented with another interesting population in which to explore vascular risk in OA and RA, when a 15 year follow up study of a prolific surgeon’s experience of knee replacements revealed that four-fifths were deceased (Loughead et al. 2008). Unlike patients who come to surgery today, the population was of almost equal numbers diagnosed with OA and RA, and all patients crossed one surgeon’s threshold for surgery and were likely to be similarly disabled at the time of the surgery. We were interested to investigate the ages and causes of death in this population.

## Methods

The population had recently been studied for outcome from the surgery (Loughead et al. 2008). The original population was of 619 patients identified from theatre records having had total knee replacements performed by one surgeon between 1980 and 1990. Records of the patients were available and were demographically updated from the Newcastle Hospitals Patient Administrative System and then we used The National Health Service Strategic Tracing Service to check if patients were still alive, and identify patients who were deceased and the date of death. The patient data was further refined, identifying the National Health Service number on all patients and the most recent known address. Information on the cause of death was requested from National Health Service Medical Research at The Information Centre for Health and Social Care who provided death certificates in December 2008.

The way in which death certificates were completed was variable with different words being used in different parts of the certificate. Similarly, descriptions of associated conditions in “Methods” may have been relevant underlying the cause of death in “Background”. It was therefore necessary for the certificates to be scrutinised by a clinician to allocate a suitable category for cause of death. The clinician was blind to the diagnosis of arthritis unless it appeared on the certificate. Deaths were allocated as primarily cardiovascular, cerebrovascular or peripheral vascular, or were allocated to another cause of death. Other causes are categorised as in Table 1. A file of what was written on the certificates and how we categorised them is available.

**Table 1:** The number of people dying of the different causes

Cause of death	OA n = 249		RA n = 246	
	Number of subjects by diagnosis	Mean age at death	Number of subjects by diagnosis	Mean age at death
	Number (%)	Years (range)	Number (%)	Years (range)
Cardiovascular	64 (25.70%)	81.14 (58–95)	65 (26.42%)	75.23 (61–95)
Cerebrovascular	21 (8.43%)	84.57 (69–93)	20 (8.13%)	77.96 (67–91)
Peripheral vascular	5 (2.01%)	77.64 (73–81)	10 (4.07%)	74.65 (48–87)
Rheumatoid arthritis	0 (0%)	0	4 (1.63%)	79.36 (73–83)
Septic	61 (24.50%)	84.22 (62–100)	62 (25.20%)	74.98 (55–92)
Cancer	27 (10.84%)	81.14 (66–99)	9 (3.66%)	75.89 (56–85)
Accident	2 (0.80%)	81.97 (76–88)	2 (0.81%)	76.05 (72–81)
Renal	6 (2.41%)	85.47 (71–96)	7 (2.85%)	75.06 (58–83)
Chest	1 (0.40%)	67.49 (0)	9 (3.66%)	74.21 (65–83)
Old age	11 (4.42%)	88.23 (80–94)	5 (2.03%)	82.00 (76–90)
Neurological	7 (2.81%)	88.67 (82–96)	2 (0.81%)	81.11 (77–86)
Gastrointestinal and liver	3 (1.21%)	80.88 (76–89)	12 (4.88%)	69.99 (49–80)
Pulmonary embolism	6 (2.41%)	83.23 (76–90)	1 (0.41)	63.07 (0)
No diagnosis	35 (14.06%)	75.49 (53–90)	38 (15.45%)	70.04 (45–82)

Number (%).

## Analysis

Ages at death were compared using the log-rank test, censoring age of patients at the study cut-off date of 1st August 2008. Competing risks analysis (Fine and Gray 1999) was used to compare the RA and OA groups in terms of vascular death; all deaths of other causes were classified as a competing risk and the analysis was adjusted for age and sex. STATA v11 was used for all analysis.

## Results

The total population was of 607: 294 OA and 313 RA. 249 of the OA (85%) and 246 of the RA (79%) subjects were deceased. Females accounted for 65% of the OA and 80% of the RA patients. 11% of the OA and 24% of the RA had bilateral knee replacements. Death certificates were found for 86% (214) of the OA and 84% (207) of the RA patients. RA appeared on the death certificates of 45 people (22%). For OA it was only 2 (1%).

The ages at operation and death for both populations were different. The OA patients had a mean age at first surgery of 70.79 compared to the RA patients at 63.58 years (p < 0.001, t test). The ages at death were similarly different with the OA patients dying at a median age of 81.97 years and the RA at 74.5 years (P < 0.001, log-rank test). OA patients came to surgery an average of 7 years older and died at a similarly older age. The patients who were identified alive (109 patients, 3 status not known) at the censored data point of 1st August 2008 had a median age for OA of 88.87 years and for RA median age of 76.56.

The causes of death for the two populations are shown in Table 1. 30% of the OA population and 28% of the RA population had died of vascular causes. Of the other causes only cancer (more common in the older OA population) looked different. Vascular causes of death are similar in OA and RA patients controlled for lower limb disability.

The ages at death for the different causes of death are also shown in Table 1. As can be seen the OA patients died at an older age than the RA patients for each cause of death. The increased age at death was consistent over all causes.

**Figure 1:**
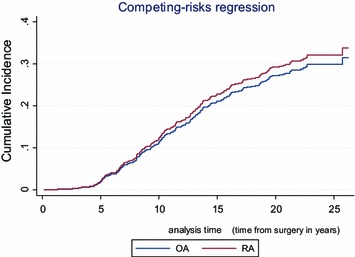
Competing risk analysis for death over time starting with the date of operation. Probability of dying a vascular death adjusted for age, disparity and competing risks.

The competing risk analysis showed that the RA subjects were at a 9% greater risk of a vascular death compared to the OA subjects (OR 1.09 95% CI 0.6–1.5), adjusted for age and sex. This was not significantly different. The cumulative incidence of death from a vascular cause over time starting with the date of operation are shown in Figure 1 for both RA and OA subjects and can be seen to be similar.

## Discussion

This population is an interesting natural experiment where substantial and almost equal numbers of OA and RA patients have been operated on and followed up by a single surgeon. All of the patients will have crossed this surgeon’s threshold for surgery which would have been based on the amount of pain and disability that the knee was causing at the time the decision was made. It is likely that a similar threshold would have been used for both populations although the upper limb disability is likely to have been greater in the RA group. The progression of the disability in the RA group is likely to have been quicker meaning that the area under the time/disability curve may have been smaller for the RA patients. Nevertheless, these patients are reasonably controlled between populations for their severe disability, allowing us to look at other factors in relation to the cause of death. No significant differences in terms of death from a vascular cause were found between the OA and RA samples, indicating that OA patients may be at a similarly high risk for cardiovascular death as RA patients, as long as they are similarly disabled.

In contrast there was a difference in age at death between the populations. OA is a condition that tends to start later in life than RA, and becomes progressively more common with age. RA on the other hand more usually comes on in middle age. Both are likely to cause increasing disability with increasing duration. If physical disability is a major factor in cardiovascular disease in these populations then it is likely to be a risk that increases with increased disability and with duration of that disability and association with disease severity has been shown (Peltomaa et al. 2002). The later onset of disease and disability in the OA population leading to increased vascular disease at a later age than RA would be expected and is consistent with this data. The increased cardiovascular disease in RA has been associated with more multisystem involvement and higher inflammation as measured by ESR and CRP (Wallberg-Jonsson et al. 2000). This increased inflammation, however, also marks for more severe disease and so these patients are likely to be more disabled as well.

The observation that the OA and RA patients died of similar causes suggests that the risk is related to things that OA and RA share. As well as disability/deconditioning this would include some treatments such as non-steroidal anti-inflammatory drugs (NSAIDs) and analgesics which both populations take. Unfortunately we do not have detailed information on the drug usage of these patients. They are all likely to have been tried on NSAIDs and analgesics prior to consideration of surgery, but it is likely to be the dose and duration of treatment that is important as shown in the coxib studies (Hippisley-Cox and Coupland 2005).

There is some support for exercise decreasing the cardiovascular risk in RA from a study looking at an education programme where exercise was encouraged (Holly et al. 2013).

In a study of cause of death in RA from a similar time period in Sweden (Kapetanovic et al. 2011), cardiovascular disease accounted for 46% compared to 20% in our population. A much bigger difference was seen for malignancy with 29% in their population and only 4% in our population. However, infection was more common in our population (25 versus 13%). Interestingly RA appeared on the death certificate in similar proportion of patients (23% in Sweden and 18% UK). OA appeared on the death certificates in less than 1% of our population.

## Limitations

There is huge variability in the words used on death certificates and in order to categorise them, arbitrary and convenient decisions have been made. A file is available. This is a retrospective observational study and so has limitations. We are limited by the data that was collected at the time and so unable to look in detail at traditional risk factors for cardio-vascular disease. We do not have detailed breakdown of drug therapies or other measures of disability. As discussed above there will be some differences in the development and effects of the disease between the diagnoses and the surgeon may have had slightly different criteria for surgery in OA and RA. The RA population was younger and this may have deterred the surgeon who would be concerned about the lifespan of the prosthesis. However, there would also be an inevitability about the future need for surgery in a patient with destructive RA that might have made the decision to operate more likely. Overall it is likely that these populations had similar lower limb disability at the time of surgery.

## Conclusion

In conclusion, the rate of vascular cause of death was similar between the two populations, controlled for lower limb disability, suggesting similarities between OA and RA that gives rise to this effect. This could be the physical disability, which was severe in both of these groups, or an effect of common treatments such as NSAIDs or analgesics. The age at death was significantly different and is therefore likely to relate to differences between OA and RA. These would include age of onset and inflammation.
